# Bidirectional risk association between adenoid hypertrophy and laryngopharyngeal reflux: A systematic meta-analysis

**DOI:** 10.1097/MD.0000000000044376

**Published:** 2025-09-12

**Authors:** Chaofan Li, Juan Ye, Shaokun Huang, Chaohua Wang, Guoping Yin

**Affiliations:** a Qinghai University Medical College, Xining, Qinghai, People’s Republic of China; b Beijing Tsinghua Chang Gung Hospital, Tsinghua University, Beijing, People’s Republic of China.

**Keywords:** adenoid hypertrophy, bidirectional comorbidity, child, laryngopharyngeal reflux, meta-analysis, neuroinflammatory pathway, pepsin-mediated inflammation

## Abstract

**Background:**

Adenoid hypertrophy (AH) and laryngopharyngeal reflux (LPR) are common, pathologically linked pediatric otolaryngological conditions; however, their causal relationship and mechanistic underpinnings are presently unclear. This study aimed to explore the association between AH and LPR in pediatric populations.

**Methods:**

Two investigators independently performed literature screening across PubMed, Web of Science, and Cochrane Library databases. Newcastle–Ottawa Scale was employed to assess the methodological quality of cohort studies, as well as Appraisal tool for Cross-Sectional Studies was applied for evaluating cross-sectional investigations. The funnel plot was used to analyze publication bias. A meta-analysis of the extracted data was conducted by using R software platform (version 4.4.2, R Foundation, Vienna, Austria).

**Results:**

A total of 18 studies (3 cross-sectional study and 15 cohort studies) were included, including 39,427 participants. Meta-analysis revealed elevated prevalence of LPR among children with AH, with a significantly increased risk of LPR in AH children (odds ratio = 10.53). Meanwhile, children underwent >1 time adenoidectomy (AT) exhibited increased risk of LPR (risk ratio = 9.43). All these data indicated LPR may play a role on AH. However, data from AT studies showed short-term symptoms of LPR could be alleviated (<1 year) after AT, but increased susceptibility to LPR (risk ratio = 2.03) was found with long-term follow-up (>10 years). These results suggested LPR was not only increase the risk of AH but also affected by AH.

**Conclusion:**

This study preliminarily validates the close association between AH and LPR. Future studies employing standardized diagnostic criteria are required to further elucidate the reciprocal relationship between AH and LPR, as the marked heterogeneity among earlier studies has primarily stemmed from variations in diagnostic methodologies.

## 1. Introduction

Adenoid hypertrophy (AH) and laryngopharyngeal reflux (LPR) constitute 2 prevalent yet pathophysiologically interconnected clinical entities in pediatric otorhinolaryngology. Compelling epidemiological data demonstrates their substantial healthcare ramifications: population-based studies reveal AH prevalence of 34.46% (n = 1446), escalating to 40% to 70% among pediatric otolaryngology outpatients (n = 3332),^[1]^ while LPR-related pathologies afflict 10% of general otolaryngology patients.^[2]^

The reciprocal relationship between AH and LPR has been extensively documented in clinical practice, yet their causal nexus remains unresolved. Notably, while LPR in pediatric populations generally presents with mild symptomatology, a significant comorbidity exists between AH and LPR. Emerging mechanistic evidence, including seminal work by Tan et al,^[3]^ posits that retrograde transport of gastroduodenal constituents (e.g., pepsin) to the nasopharynx via LPR may initiate adenoid inflammation. Conversely, AH-induced airway obstruction potentially aggravates reflux through perturbations in intrathoracic pressure dynamics,^[4]^ though contradictory findings persist across studies.^[5]^ Moreover, current fragmented research paradigms lack sufficient power to establish definitive correlations. Although minimizing general anesthesia in pediatric surgical interventions remains a clinical imperative, isolated therapeutic approaches such as topical corticosteroids demonstrate limited efficacy in addressing pediatric AH. This investigation systematically evaluates the AH–LPR interaction through quantitative methodologies, providing preliminary validation of the critical need to integrate anti-reflux strategies with conventional AH management protocols.

## 2. Methods

### 2.1. Registration

This study was registered on PROSPERO with the registration number CRD420251054260.

### 2.2. Data sources

Our systematic review involved a comprehensive literature search conducted in the PubMed, Web of Science, and Cochrane Library databases. The search criteria included articles published between 2000 and 2025, available globally in either English or Chinese, encompassing randomized controlled trials, observational studies, and cross-sectional studies. The search strategy incorporated a combination of free-text keywords and controlled vocabulary (MeSH terms: “adenoids/hypertrophy” and “laryngopharyngeal reflux”) connected via Boolean operators. Study selection proceeded through a rigorous 3-stage screening methodology comprising initial automatic deduplication, independent blinded evaluation of titles and abstracts by reviewers, and subsequent full-text assessment.

The methodological quality of included studies was systematically assessed using standardized tools, with cross-sectional studies evaluated via the Appraisal tool for Cross-Sectional Studies checklist (scores > 14/20 indicating acceptable quality) and observational studies assessed using the Newcastle–Ottawa Scale (acceptable quality defined as Newcastle–Ottawa Scale scores > 5/9). Randomized controlled trials provided insufficient data for quantitative meta-analysis and thus were qualitatively synthesized. To enhance comprehensiveness, additional pertinent studies were sourced via manual citation tracking from the reference lists of retrieved articles, thereby ensuring thorough coverage of the pathophysiological associations between AH and LPR.

Studies identified were eligible for inclusion if they fulfilled all of the following criteria: enrolled pediatric subjects aged ≤ 18 years; clearly diagnosed AH, confirmed by nasopharyngoscopy or X-ray imaging; and definitively established LPR diagnosis via pH monitoring, validated symptom scoring scales, pepsin detection assays, or previously documented clinical diagnostic coding. Conversely, studies meeting any one of the following exclusion conditions were excluded: participants presenting with craniofacial or upper airway congenital malformations or developmental abnormalities; documented congenital gastrointestinal disorders, such as Crohn disease; pediatric subjects undergoing an acute inflammatory episode of the upper respiratory or gastrointestinal tract; research exclusively involving adult populations; and studies that exclusively reported combined adenotonsillectomy procedures without distinctly separate data for adenoidectomy (AT) outcomes (Tables 1–4).

**Table 1:** PECO framework.

Stage	P (Population)	E (Exposure)	C (Comparator)	O (Outcome)
Stage 1: epidemiological association	Children under 18 years of age diagnosed with AH	Confirmed diagnosis of AH	Healthy children without AH or general pediatric population	Prevalence of LPR and associated risk (OR) of developing LPR
Stage 2: postoperative reflux improvement	Children diagnosed with AH who underwent AT	AT	Children with unoperated AH or preoperative baseline data	Improvement rate of postoperative LPR symptoms (RR) (short-term: <1 year; long-term: more than 10 years)
Stage 3: reoperation risk in AH Patients with LPR	Children undergoing revision adenoidectomy	Revision AT	Children who underwent a single AT procedure only	Risk of LPR-associated revision surgery (RR)

AH = adenoid hypertrophy, AT = adenoidectomy, LPR = laryngopharyngeal reflux, OR = odds ratio, RR = risk ratio.

**Table 2:** Raw data from META-analysis.

Study	Event	Total	Ctrl_event	ctrl_Total	Country	Age_range (years)	Design	Detection	Quality assessment	
Johnston et al^[6]^	9	212	27	8620	New Zealand	6.70 ± 0.47	Cohort study	Clinical coding	**7**	
Wasilewska et al^[5]^	8	19	4	38	Poland	6.9 ± 0.5	Cohort study	24-pH	**7**	
Monroy et al^[7]^	28	29	2	29	United States	7.76 ± 4.0	Cohort study	24-pH	**6**	
Dearking et al^[8]^	638	8245	314	8245	United States	6.7 ± 3.7	Cohort study	Clinical coding	**6**	
Shatz^[9]^	0	24	21	24	Israel	NA (0.83)	Cohort study	24-pH	**6**	
Sagar et al^[4]^	2	49	10	49	India	NA (6)	Cohort study	24-pH	**6**	
Huang et al^[10]^	52	71	0	0	China	NA (3–12)	Cross-sectional study	RFS + RSI	*16/20*	
Lin et al^[11]^	149	190	0	0	China	5.3 ± 2.0 (3–13)	Cohort study	Pepsin	**7**	
Zhou et al^[12]^	8	43	0	0	China	NA (2–10)	Cohort study	Pepsin	**7**	
Marzouk et al^[13]^	60	207	0	0	United States	NA (0.5–12)	Cohort study	RFS + RSI	**4**	
Shatz^[9]^	21	24	0	0	Israel	NA (≤1)	Cohort study	24-pH	**5**	
Chorney et al^[14]^	1683	21,232	0	0	United States	4.4 ± 0.1	Cross-sectional study	Clinical coding	*14/20*	
Harris et al^[15]^	0	21	0	12	Australia	NA (2–10)	Cross-sectional study	Pepsin	*16/20*	
Keles et al^[16]^	14	30	1	12	Turkey	6.5 ± 2.4 (3–12)	Cohort study	24-pH	**6**	**NOS**
Melake et al^[17]^	18	32	4	40	Saudi Arabia	NA (2–14)	Cohort study	HP	**7**	*AXIS*
Saki et al^[18]^	21	84	18	91	Iran	NA (1–10)	Cohort study	HP	**7**	
Tumgor et al^[19]^	32	44	0	20	United States	NA (3–12)	Cohort study	RFS + RSI	**5**	
Yilmaz et al^[20]^	12	18	1	20	Turkey	7.85 ± 2.9	Cohort study	24-pH	**7**	

Bold values denote quality scoring utilizing the NOS for cohort studies, while italic values indicate quality scoring based on the AXIS for cross-sectional studies. AXIS = Appraisal tool for Cross-Sectional Studies, NOS = Newcastle–Ottawa Scale, RFS = Reflux Finding Score, RSI = Reflux Symptom Index.

**Table 3:** NOS quality evaluation tool.

Study	Year	Selection	Comparability	Outcome	Score
Keles et al^[16]^	2005	★★	★	★★★	6
Lin et al^[11]^	2004	★★★	★	★★★	7
Melake et al^[17]^	2012	★★★	★	★★★	7
Saki et al^[18]^	2014	★★★	★	★★★	7
Tumgor et al^[19]^	2021	★★	★	★★	5
Yilmaz et al^[20]^	2005	★★★	★	★★★	7
Zhou et al^[12]^	2023	★★★	★	★★★	7
Marzouk et al^[13]^	2012	★★	★	★★	5
Shatz^[9]^	2004	★★	★	★★★	6
Johnston et al^[6]^	2017	★★★	★	★★★	7
Wasilewska et al^[5]^	2011	★★★	★	★★★	7
Monroy et al^[7]^	2008	★★★	★	★★	6
Dearking et al^[8]^	2012	★★★	★	★★	6
Sagar et al^[4]^	2020	★★	★	★★★	6

NOS = Newcastle–Ottawa Scale.

**Table 4:** AXIX quality evaluation tool.

Study	Year	Q1	Q2	Q3	Q4	Q5	Q6	Q7	Q8	Q9	Q10	Q11	Q12	Q13	Q14	Q15	Q16	Q17	Q18	Q19	Q20	Score
Harris et al^[15]^	2009	1	1	1	1	1	0	1	1	1	1	1	−1	0	0	1	1	1	1	1	1	15
Huang et al^[10]^	2018	1	1	0	1	1	1	0	1	1	1	1	1	0	0	1	1	1	1	1	1	16
Chorney et al^[14]^	2021	1	1	0	1	1	0	0	1	1	1	1	1	0	0	1	1	1	1	0	1	14

1, yes; 0, do not know; − 1, no. AXIS = Appraisal tool for Cross-Sectional Studies.

Eligibility screening and abstract evaluation processes were independently carried out by 3 reviewers (Li, Wang, and Ye), with any disagreements resolved through consensus-based discussion.

### 2.3. Diagnostic criteria

In our selection of literature, we adopted commonly used standard diagnostic methods for identifying children with (AH), including nasopharyngeal endoscopic grading and the adenoidal–nasopharyngeal ratio determined by lateral neck radiography.^[21]^ For endoscopic assessment, we utilized the grading criteria proposed by Parikh et al: Grade 1 – adenoid tissues that do not contact adjacent structures at rest; Grade 2 – adenoid tissues contacting the torus tubarius; Grade 3 – adenoid tissues reaching the vomer; and Grade 4 – adenoid tissues in contact with the soft palate.^[22]^

For screening and confirmation of LPR within the reviewed literature, we recognized 3 mainstream diagnostic methods: the combined application of Reflux Symptom Index and Reflux Finding Score, multichannel intraluminal impedance–24-hour pH monitoring, and the detection of pepsin in saliva. Both Reflux Symptom Index and Reflux Finding Score scales possess robust validity and reliability,^[23,24]^ making them effective tools for initial diagnosis and therapeutic evaluation in LPR disease. Multichannel impedance–24-hour pH monitoring technology effectively captures nonacidic reflux events, which traditional pH monitoring often misses, thus playing a critical role in the accurate diagnosis of LPR.^[25]^ Additionally, pepsin detection leverages the role of pepsin as a digestive enzyme whose elevated concentrations in saliva act as biomarkers signaling reflux from gastric contents to the laryngopharynx.^[26]^

### 2.4. Data extraction

Data extraction encompassed 3 distinct domains: evaluation methodologies (participant assessment procedures, geographic distribution, and observational study designs incorporating both cohort and cross-sectional frameworks); demographic characteristics (criteria for age stratification and diagnostic validation protocols pre- and post-intervention); and specified meta-analytic objectives, namely: the prevalence of AH–LPR comorbidity, rates of symptomatic improvement of LPR following adenotonsillectomy, and incidence of LPR among pediatric patients undergoing repeat adenotonsillectomy (AT). Extraction was undertaken independently by 2 reviewers (Li and Huang), with any discrepancies resolved via collaborative reassessment and consensus discussion.

### 2.5. Statistical analysis

This study performed a meta-analysis integrating datasets from 3 time points. Considering heterogeneity among studies, either random-effects or fixed-effects models were applied to calculate pooled estimates with corresponding 95% confidence intervals (CIs). In the first-stage meta-analysis, sensitivity analysis using the leave-one-out approach identified diagnostic methodology as a major source of heterogeneity; thus, subgroup analyses were conducted based on different diagnostic criteria. Small-study effects were assessed via Egger and Begg tests, and publication bias was evaluated and adjusted using the trim-and-fill method. Subgroup analyses were similarly performed in the second and third stages. All statistical analyses were conducted using R software (version 4.4.2) under the supervision of 2 researchers (Yin and Li).

## 3. Results

### 3.1. Study characteristics

A triage of 29 potentially eligible publications was identified through title/abstract screening from an initial pool of 162 studies. Following meticulous full-text evaluation, 18 investigations ultimately met inclusion criteria, encompassing 39,427 patients. The comprehensive selection workflow is delineated in the preferred reporting items for systematic reviews and meta-analyses 2020-compliant flow diagram (addition: preferred reporting items for systematic reviews and meta-analyses flow diagram).

### 3.2. Meta-analysis findings

These 18 studies methodologically divided the analytical continuum into 3 stratified stages.

#### 3.2.1. Stage 1: epidemiological association

**Figure 1.**
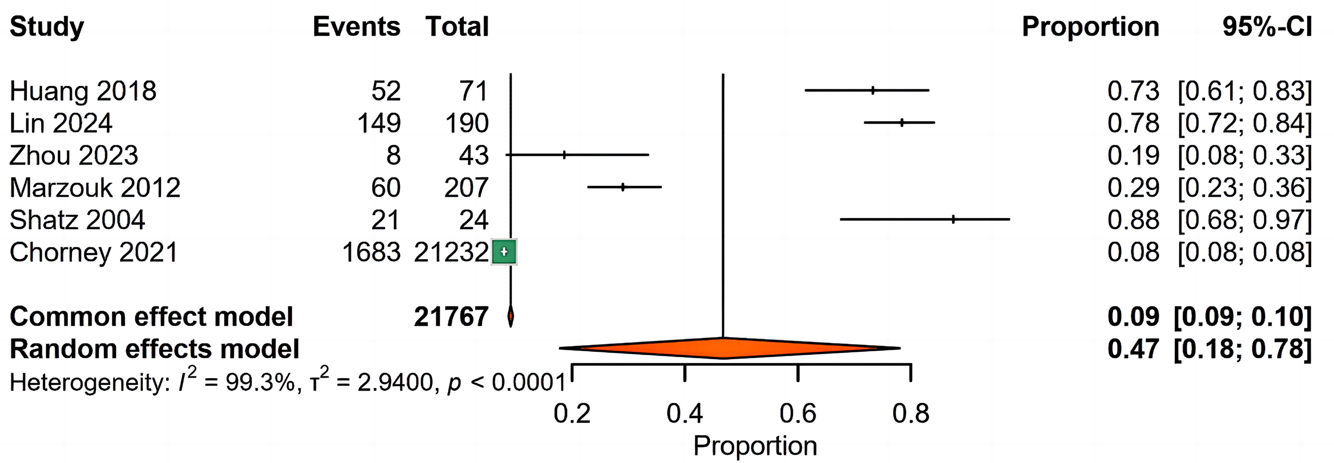
A single-arm meta-analysis was performed on the 6 datasets included.

Our preliminary analysis comprised a meta-analysis of 6 observational studies (n = 21,767), which demonstrated substantial heterogeneity (I² = 99.3%, τ² = 2.94). Therefore, a random-effects model was utilized, revealing a pooled prevalence of LPR at 47% (95% prediction interval: 18%–78%) among pediatric subjects diagnosed with AH. Given this significant heterogeneity, we conducted a leave-one-out sensitivity analysis, which indicated consistent directional prevalence estimates (range: 0.37–0.59), although specific studies notably influenced the overall results. Specifically, exclusion of Chorney et al ^[14]^ elevated the estimated prevalence to 59% (95% CI: 31%–82%), while removal of Zhou et al^[12]^ lowered it to 37% (95% CI: 14%–68%). Furthermore, iterative analyses demonstrated 86% concordance with the primary analysis’ 95% CIs, indicating that methodological differences rather than isolated outlier influences primarily explained the observed heterogeneity (Figs. 1 and S1, Supplemental Digital Content, https://links.lww.com/MD/P909).

**Figure 2.**
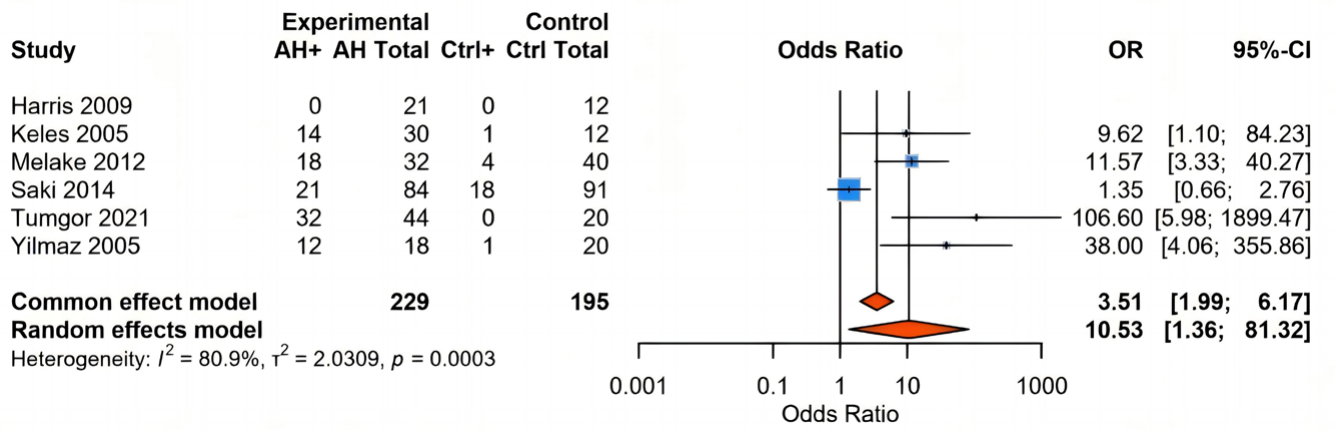
A two-arm meta-analysis was conducted on the 6 datasets included.

Subsequent analyses of 6 pediatric studies (n = 424) evaluated whether the prevalence of LPR among pediatric patients with AH exceeds that observed in general pediatric populations. Employing a random-effects meta-analysis (I² = 80.9%, τ² = 2.03), we observed a significantly increased risk of LPR in patients with AH compared to their non-AH counterparts (odds ratio [OR] = 10.53, 95% CI: 1.36–81.32, *P* = .024). Stratified analyses demonstrated clear methodological dependency, with consistent and substantial risk elevations identified in studies using 24-hour pH monitoring to quantify pharyngeal acid exposure (risk ratio [RR] = 8.68; 95% CI: 2.29–32.87; I² = 0%, *P* = .533). Conversely, studies employing Helicobacter pylori-focused diagnostic indicators manifested attenuated yet significant associations accompanied by greater heterogeneity (RR = 2.01; 95% CI: 1.27–3.18; I² = 85.2%, *P* = .0093). Additionally, leave-one-out sensitivity analyses of heterogeneous datasets consistently supported the directional robustness of estimates (range in RR: 4.00–7.31). Specifically, the exclusion of Saki et al’s cohort decreased relative risk estimates from RR = 7.31 (95% CI: 3.41–15.69) to RR = 4.00 (95% CI: 1.21–13.24) after omitting Tumgor et al’s study, highlighting the influence of individual data sources on pooled outcomes (Figs. 2 and S3, Supplemental Digital Content, https://links.lww.com/MD/P909).

#### 3.2.2. Stage 2: postoperative reflux improvement

**Figure 3.**
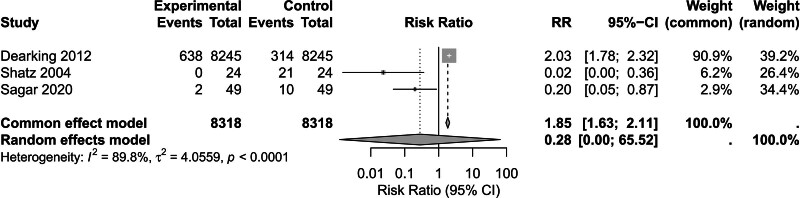
Prevalence of LPR following AT in pediatric patients diagnosed with AH complicated by LPR.

A secondary analysis involving 8318 patients was conducted to evaluate postoperative improvement in reflux symptoms, and the pooled RR using a random-effects meta-analysis was 2.03 (95% CI: 1.78–2.32; I² = 89.8%; τ² = 4.0559). Subsequent subgroup stratification, performed after excluding the study by Dearking et al^[8]^ due to its significantly prolonged follow-up (>10 years), revealed marked temporal heterogeneity. Specifically, cohorts assessed using 24-hour pH monitoring and short-term follow-up (<1 year) demonstrated a considerably greater improvement, reflected by a fixed-effects RR of 0.08 (95% CI: 0.02–0.28; *P* = .001; I² = 45.5%; τ² = 1.0530). Notably, the exclusion of the Dearking study led to a substantial reduction in between-study heterogeneity, accounting for approximately 78% of variance (with τ² decreasing from 8.95 to 1.05). These findings collectively indicate significant clinical heterogeneity arising from differences in diagnostic methodologies and underscore the potential impact of cumulative measurement errors on the assessment of long-term outcomes (Figs. 3 and S5, Supplemental Digital Content, https://links.lww.com/MD/P909).

#### 3.2.3. Stage 3: reoperation risk in AH patients with LPR

**Figure 4.**
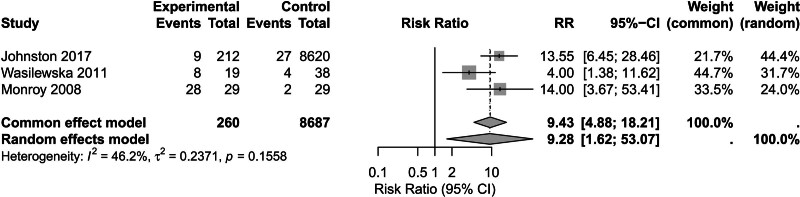
Comparison of LPR prevalence in AH pediatric patients undergoing secondary AT versus those undergoing primary AT.

The third-stage analysis demonstrated that pediatric patients who underwent revision AT exhibited a significantly elevated risk of LPR compared to the primary surgery group (n = 8947), with a fixed-effects model yielding a RR of 9.43 (95% CI: 4.88–18.21; *P* < .001; I² = 46.2%; τ² = 0.24). Subgroup stratification further uncovered inherent methodological heterogeneity: the subgroup utilizing 24-hour pH monitoring showed an RR of 8.29 (95% CI: 3.51–19.54; I² = 51.4%; τ² = 0.40), leading to a widening of CIs (Figs. 4 and S6, Supplemental Digital Content, https://links.lww.com/MD/P909).

### 3.3. Publication bias

The assessment of publication bias in the single-arm meta-analysis revealed inconsistent outcomes: While Egger test demonstrated statistical significance (*P* = .037), suggesting potential bias, Begg test failed to reach significance (*P* = .851). Subsequent sensitivity analysis excluding studies with disproportionately large sample sizes showed that Egger test no longer reached statistical significance (*P* = .12), indicating that the observed bias might primarily stem from extreme sample size discrepancies among included studies. This differential sensitivity between statistical tests underscores the importance of methodological triangulation when evaluating potential bias in meta-analytic syntheses (Fig. S2, Supplemental Digital Content, https://links.lww.com/MD/P909).

In a subsequent two-arm meta-analysis, 3 distinct methodologies were employed to validate publication bias: Egger test demonstrated nonsignificant results across all approaches (meta package: *P* = .1857; linear regression: *P* = .889; metafor package: *P* = .5584). Furthermore, Begg test corroborated these findings (*P* = .719), indicating no substantial publication bias. Notably, trim-and-fill analysis suggested potential undetected small-scale negative studies, implying possible overestimation of the effect size (OR = 10.53, 95% CI). This methodological triangulation reveals that while conventional tests show statistical robustness, the observed OR might be inflated due to selective non-publication of negative findings from underpowered trials (Fig. S4, Supplemental Digital Content, https://links.lww.com/MD/P909).

## 4. Discussion

### 4.1. Metadata section

This 3-stage meta-analysis sheds light on the intricate association between LPR and AH in pediatric patients. Preliminary results consistently demonstrated robust correlations across all analyzed timeframes: notably higher LPR prevalence among children with AH (OR = 10.53), significant short-term improvement in LPR symptoms after adenotonsillectomy (RR = 0.08), and increased recurrence rates of LPR prompting revision surgery (RR = 8.29). Taken together, these stratified findings underscore a bidirectional, clinically nuanced interplay rather than a straightforward causal linkage. Furthermore, considerable heterogeneity in outcomes observed across varying postoperative periods emphasizes the need for prospective longitudinal studies to more accurately elucidate the temporal relationship and pathophysiological complexity between LPR and AH.

The Phase I meta-analysis revealed substantial heterogeneity (I² = 99.3%), primarily arising from evolving diagnostic protocols rather than isolated outlier effects; this finding was substantiated through iterative sensitivity analyses, demonstrating an 86% consistency rate. Stratified risk quantification further exposed significant methodological limitations inherent to current diagnostic paradigms. Specifically, studies employing 24-hour pH monitoring exhibited a substantially higher risk amplification (RR = 8.68) relative to those employing Helicobacter pylori-focused approaches (RR = 2.01), suggesting that existing heterogeneous diagnostic criteria fail to comprehensively capture the complex pathophysiological landscape of LPR–AH.

The Phase II analysis indicated that adenotonsillectomy initially provided substantial protection against reflux (RR = 0.08); however, this protective efficacy notably diminished over time (from RR = 8.95 to RR = 1.05 after accounting for the exclusion of the Dearking effect). Such a temporal trend indicates that the resolution of mechanical obstruction may temporarily obscure underlying persistent mucosal inflammation.

Phase III findings demonstrated a significantly elevated risk (9.43-fold increase) of secondary AT among pediatric patients with AH who tested positive for LPR, thus clearly establishing the etiological significance of LPR. Notably, moderate heterogeneity (I² = 51.4%) was observed in terms of effect size within the pH-monitoring subgroup, suggesting possible inconsistencies in diagnostic criteria for pediatric LPR diagnosis based on pH monitoring methodologies.

### 4.2. Advances in pathogenesis of AH–LPR

The present investigation confirmed that both 24-hour pH monitoring and histopathological evaluation exhibit low heterogeneity, thereby allowing for a more precise interpretation of diagnostic outcomes. However, significant limitations and impracticality persist when implementing these methods for LPR screening in pediatric clinical practice. The primary constraints include: the invasive nature of 24-hour pH monitoring renders it unacceptable for most pediatric patients and their caregivers; despite superior diagnostic performance compared to symptom-based scoring systems (number needed to test = 5 vs 8),^[27,28]^ its suboptimal detection capability for weakly acidic reflux events (pH 4–7) – which constitute 30% to 68% of total reflux burden^[29]^ – reveals a critical mechanistic blind spot. Specifically, conventional pH monitoring fails to detect 73% of nonacidic reflux episodes in proton pump inhibitor (PPI)-resistant populations – a diagnostic gap mechanistically aligned with the 40% PPI nonresponse rate attributed to persistent TLR4 activation at pH > 4.^[30,31]^ Pathophysiologically, pH-insensitive mucosal immune pathways (e.g., TLR4-mediated neurogenic inflammation) may play a predominant role in LPR pathogenesis.^[32]^

Furthermore, evidence suggests that the laryngopharyngeal manifestations of pediatric LPR may not be predominantly mediated by acidic components, based on the following observations: reflux symptom and sign scores in children were relatively low; PPI therapy demonstrated suboptimal efficacy; and a significant proportion of pepsin was detected in the oral cavities of children with AH and comorbid LPR. This hypothesis is further corroborated by recent findings validating the acid-pepsin synergy mechanism in LPR-driven oropharyngeal injury, wherein acid exposure suppresses mucins (MUC2/MUC5AC↓36%–72%).^[33]^ and pepsin-triggered epithelial breach activate NLRP3 inflammasomes (*R* = 0.55, *P* < .01), propelling IL-1β/TNF-α storms that recruit CD4 + T cells (β = 0.62, *P* = .007) and hyperplastic lymphoid clusters.^[3]^ Concurrently, MMP-7-driven E-cadherin degradation (↑72% activity) and NF-κB-mediated cytokine polarization (TNF-α↑3.5 × vs. IL1RN↓67%)^[34]^ disrupt mucosal repair, perpetuating inflammatory injury.^[34–36]^ These findings collectively suggest that during the pathogenesis of LPR, a series of inflammatory responses potentially mediated by pepsin may be the primary culprit underlying the development of AH.

To investigate the association between AH and LPR, some studies have also focused on elucidating the potential mechanisms by which AH may influence the occurrence of LPR. Current evidence suggests that adenotonsillar hypertrophy may induce LPR through mechanical obstruction, manifesting as reduced hyoid vertical distance (Δ = 3.2 mm; *P* = .004), diminished total airway volume (Δ = 4.1 cm³; *P* = .001), and constricted retropalatal space (Δ = 2.7 cm³; *P* = .0001). These anatomical alterations generate pathological negative intraesophageal pressure (*P* < .01),^[37]^ thereby exacerbating pharyngeal reflux. This hypothesis aligns directionally with our clinical observation of transient LPR improvement post-AT (RR = 0.08), yet paradoxically fails to explain the elevated long-term postoperative LPR incidence compared to normative populations (RR = 2.03).

The pathophysiological interplay between AH and LPR may involve mechanisms extending beyond this singular pathway. Notably, childhood obstructive sleep apnea (OSA) frequently originates from AH. Despite surgical intervention, 15.4% of cases persist with OSA within 4 months post-adenotonsillectomy.^[38]^ Furthermore, OSA patients commonly exhibit concomitant airway neural dysfunction. Importantly, emerging evidence demonstrates that neural fibers governing both respiratory and esophageal functions ultimately synapse within the nucleus tractus solitarius – projections that have been shown to originate from embryonic C-fiber lineages.^[39,40]^ In OSA, elevated loop gain exacerbates pharyngeal instability through hypoxemia-induced neuromuscular dysfunction (observed in ~36% of OSA cases) and inspiratory negative pressure gradients.^[41]^

These pathophysiological mechanisms indicate a possible comorbid relationship between OSA and LPR, whereby interactive neural influences compromise the structural integrity of the gastroesophageal junction, lowering the pressure thresholds of the lower esophageal sphincter and the upper esophageal sphincter, and consequently facilitating reflux events. Recurrent reflux-induced chronic inflammation might establish a self-perpetuating cycle, exacerbating the progression of AH. These findings suggest a complex bidirectional interaction between AH and LPR, a hypothesis that requires further comprehensive investigation.

### 4.3. Clinical insights and future directions

Currently, reflux diagnosis primarily relies on pH/multichannel intraluminal impedance–24-hour pH monitoring; however, its clinical application poses notable limitations, including invasiveness, patient discomfort, high costs, and the necessity for specialized interpretation. Its role in diagnosing pediatric LPR has therefore been increasingly questioned. There is an urgent clinical need for a rapid, noninvasive diagnostic method utilizing specific biomarkers, such as salivary pepsin detection,^[42]^ to improve individualized therapeutic strategies for children with AH.

## 5. Limitations

### 5.1. Diagnostic heterogeneity

High variability in LPR definitions (pH thresholds, pepsin assays) and age ranges (1–18 years) contributed to extreme heterogeneity (I²=99.3%), resulting in unreliable pooled prevalence estimates (18%–78% prediction interval).

### 5.2. Publication bias

While Egger and Begg tests demonstrated no significant publication bias (*P* > .05), funnel plot asymmetry revealed the absence of small-scale studies with negative outcomes, suggesting potential overestimation of effect magnitudes.

### 5.3. Subgroup power limitations

Critical analyses (e.g., pH-monitored cohorts) included only 2 studies, producing unstable RRs (RR = 0.08, 95% CI: 0.02–0.28) that require validation in larger samples.

### 5.4. Long-term data constraints

Postoperative recurrence risks relied on a single study with incomplete adjustment for confounders (e.g., weight gain, allergens) over > 10-year follow-up.

### 5.5. Mechanistic evidence gaps

The proposed neuroinflammatory mechanisms lack direct human evidence (e.g., histopathology/imaging), necessitating translational validation.

## 6. Conclusion

In this study, through a synthesis of previous research on AH and LPR, we preliminarily confirmed a close association between these 2 conditions. Given the current lack of universally accepted, effective diagnostic criteria for LPR, further studies utilizing standardized diagnostic methods and larger samples are needed to clarify the interactive relationship between AH and LPR.

## Acknowledgments

Special gratitude is extended to our esteemed colleagues at the Affiliated Hospital of Qinghai University for their invaluable support.

## Author contributions

**Data curation:** Chaofan Li, Juan Ye, Chaohua Wang.

**Formal analysis:** Chaofan Li.

**Project administration:** Chaofan Li, Guoping Yin.

**Resources:** Shaokun Huang.

**Software:** Shaokun Huang, Chaohua Wang.

**Writing – original draft:** Chaofan Li, Juan Ye, Shaokun Huang, Chaohua Wang.

**Writing – review & editing:** Chaofan Li.

## Supplementary Material


