# Supplementary material for: Bidirectional risk association between adenoid hypertrophy and laryngopharyngeal reflux: A systematic meta-analysis
Source: Medicine (Baltimore). 2025 Sep 12;104(37):e44376. doi: 10.1097/MD.0000000000044376 (PMC12440550; doi:10.1097/MD.0000000000044376)
Supplement: Supplementary file 1 [file medi-104-e44376-s001.pdf]

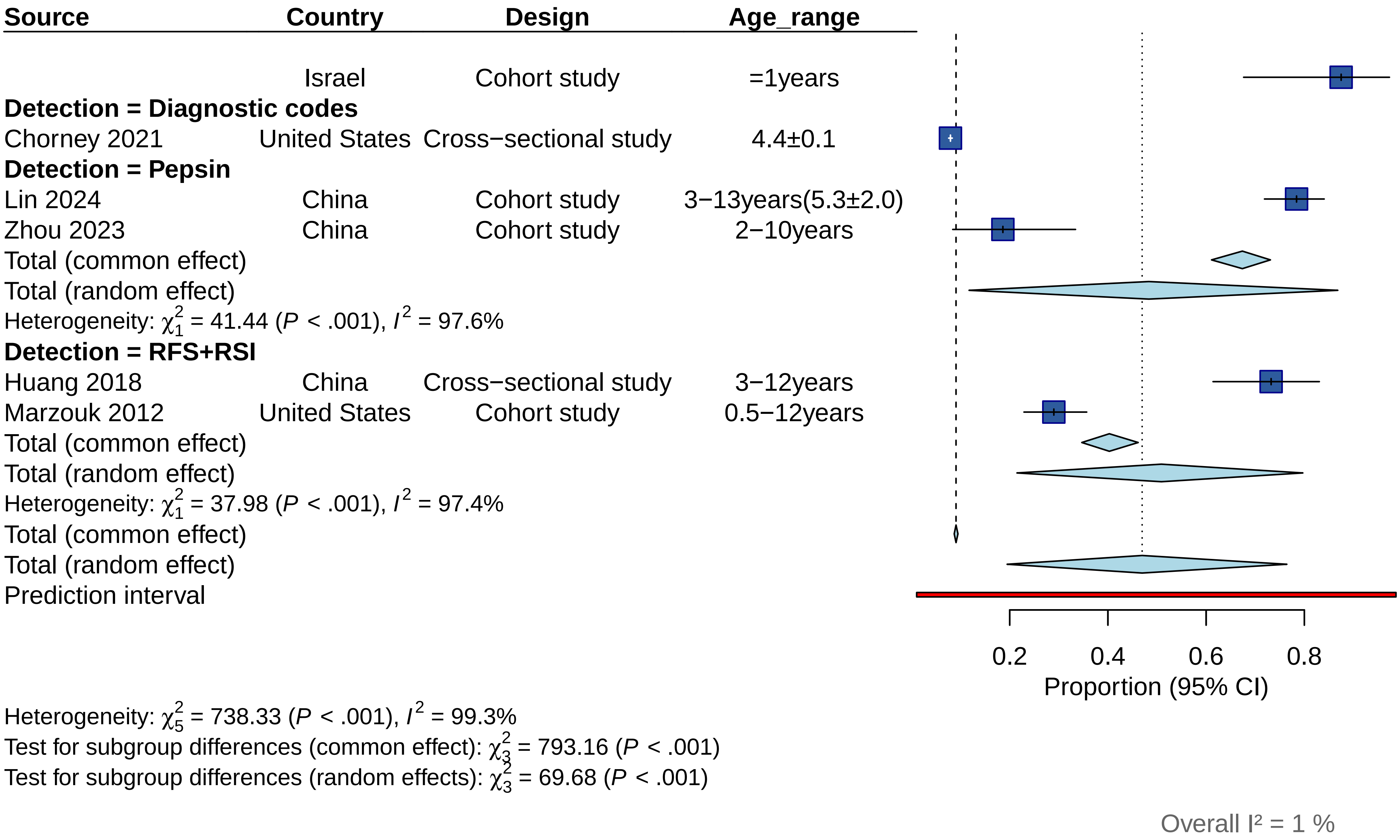

Forest plot of subgroup analysis in single-arm rate meta-analysis

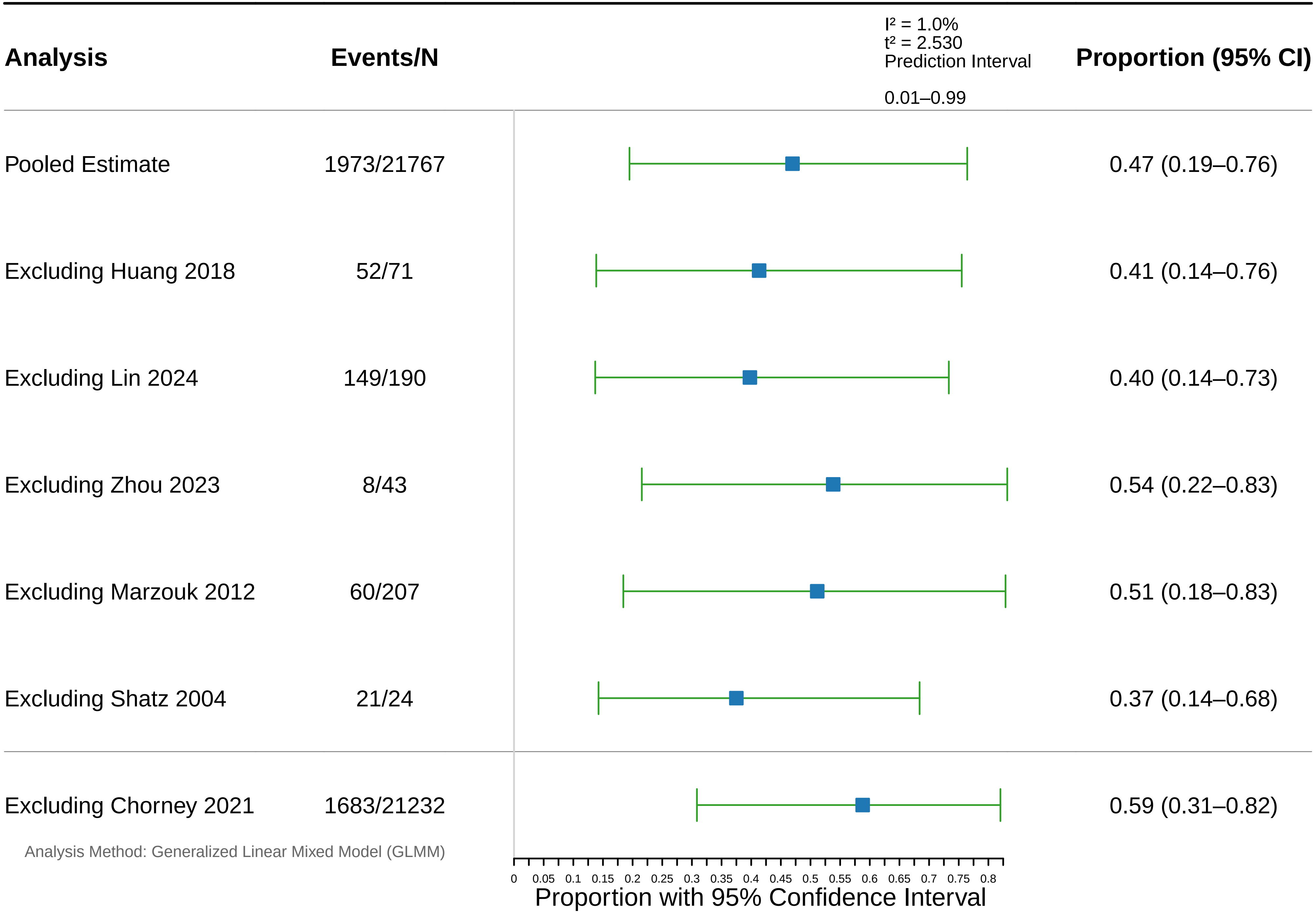

Sensitivity analysis of single-arm rate meta-analysis (leave-one-out method)

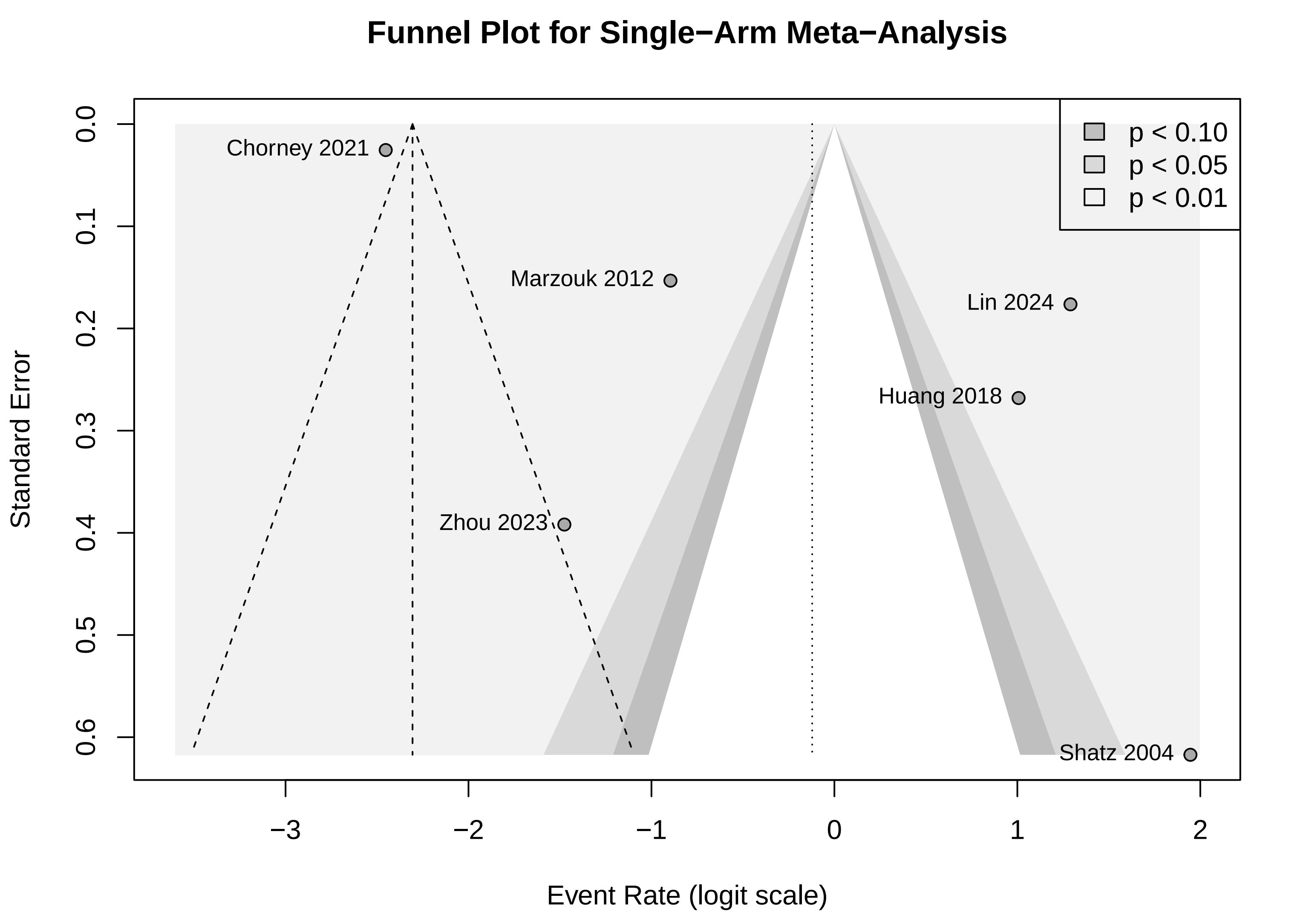

Funnel Plot for Single−Arm Meta−Analysis
Chorney 2021
Marzouk 2012
Lin 2024
Huang 2018
Zhou 2023
Shatz 2004
p < 0.10
p < 0.05
p < 0.01
Standard Error
0.0
0.1
0.2
0.3
0.4
0.5
0.6
−3
−2
−1
0
1
2
Event Rate (logit scale)

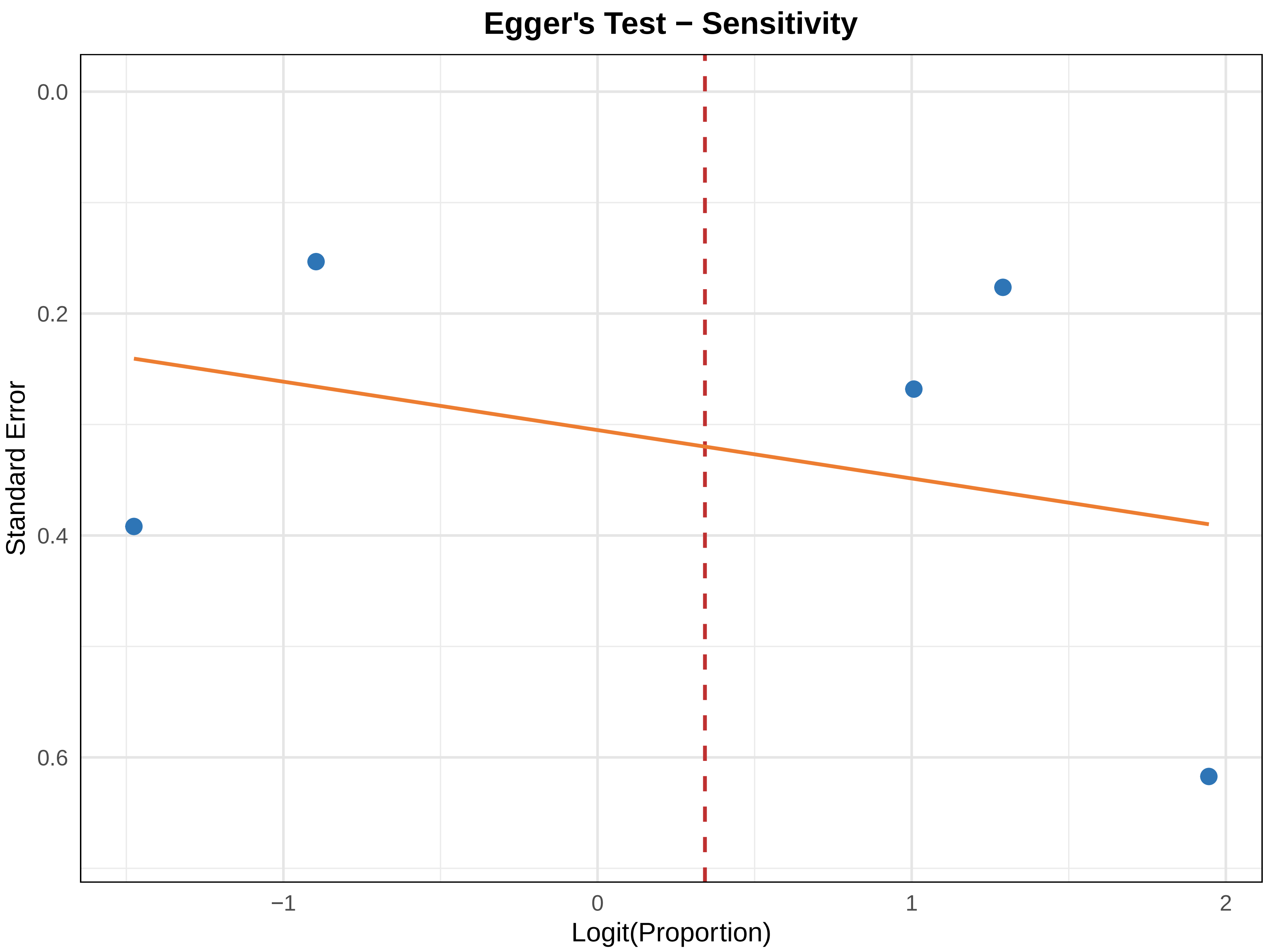

Sensitivity analysis of Egger's test for single-arm rate studies

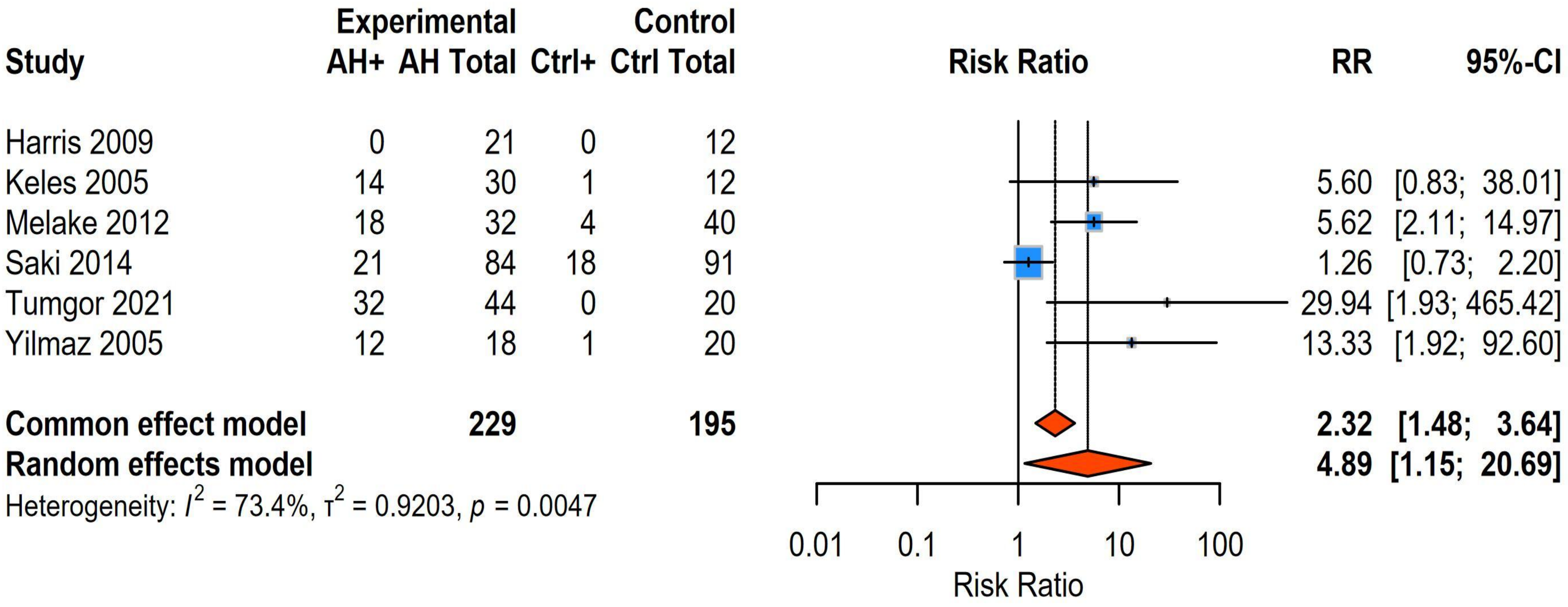

Forest plot of two-arm rate meta-analysis（RR）

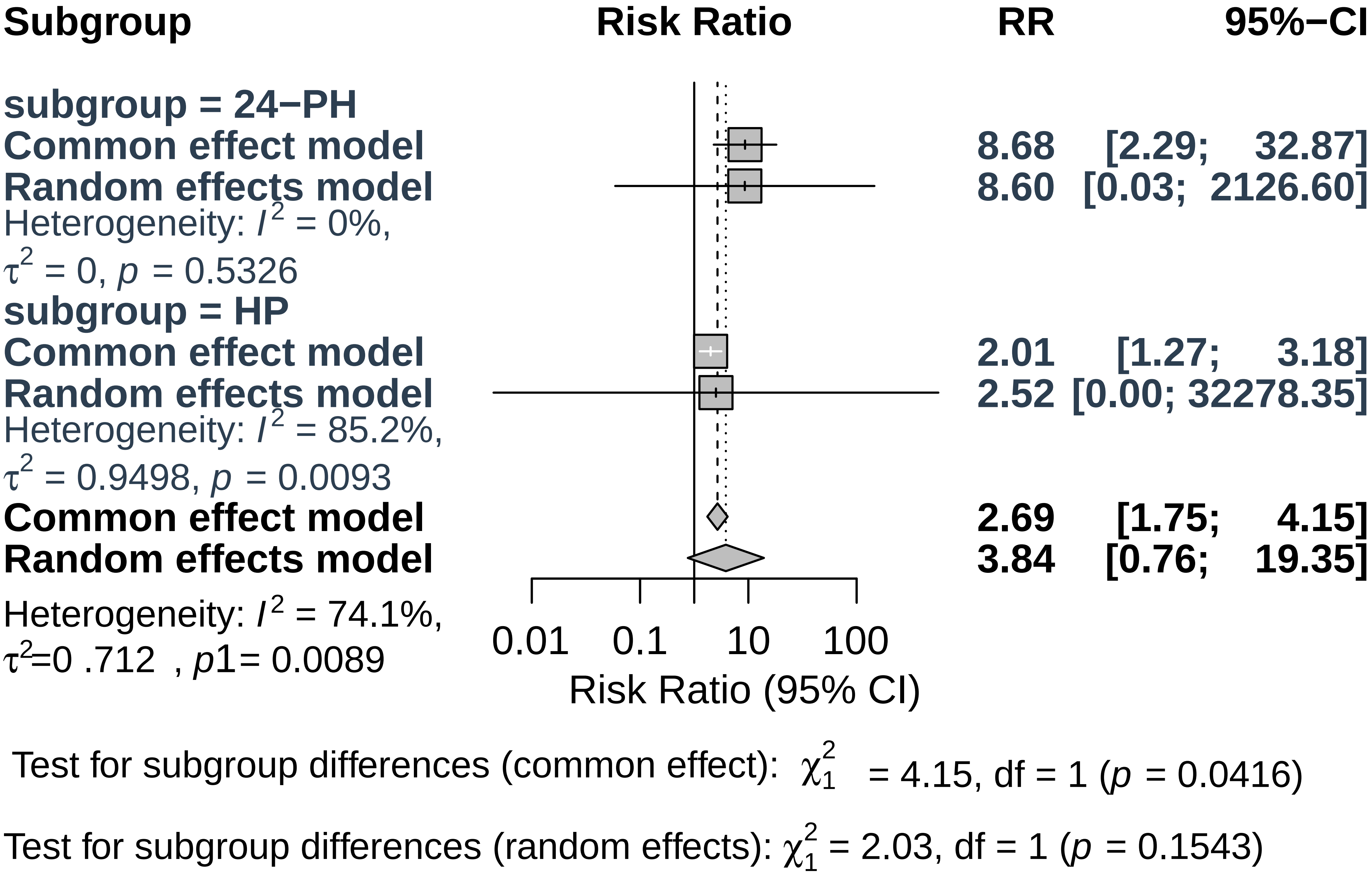

Forest plot of subgroup analysis in two-arm rate meta-analysis

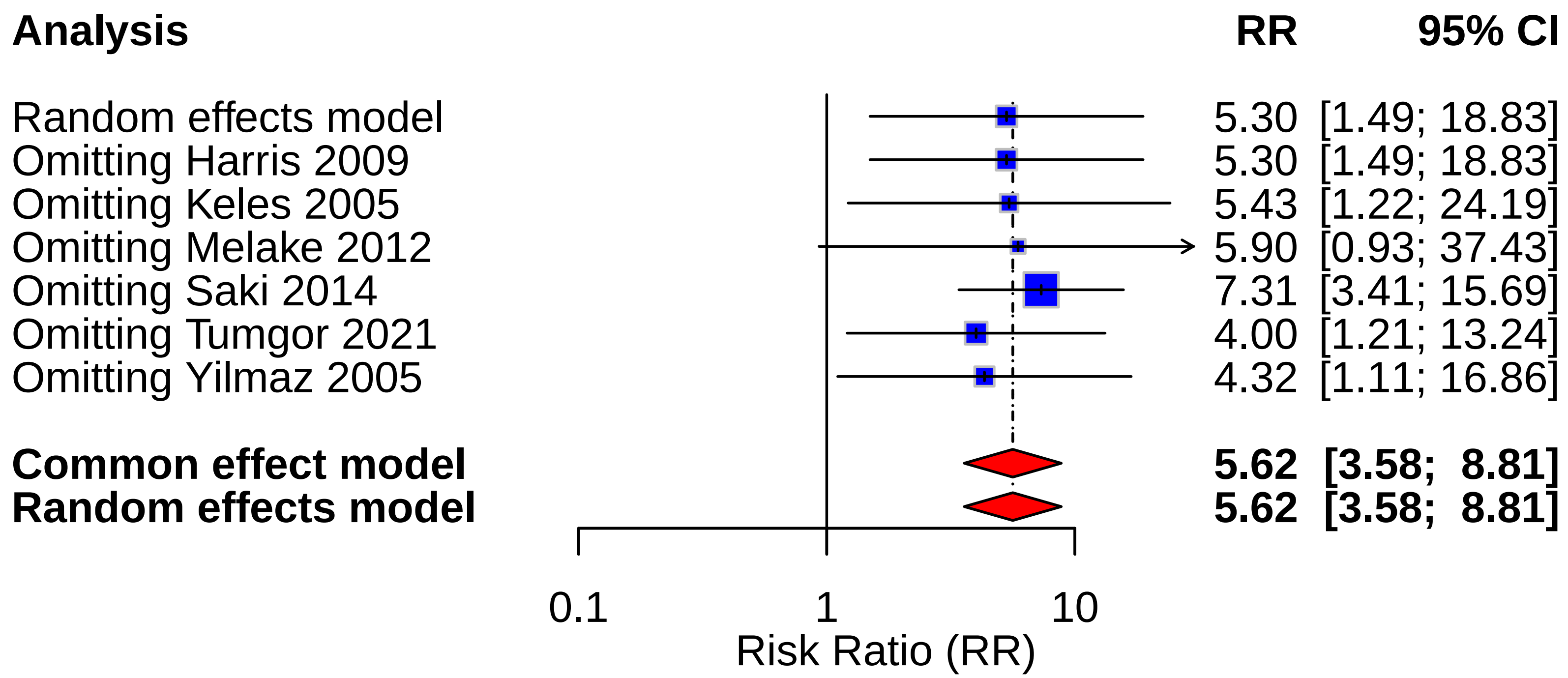

Sensitivity analysis of two-arm rate meta-analysis (leave-one-out method)

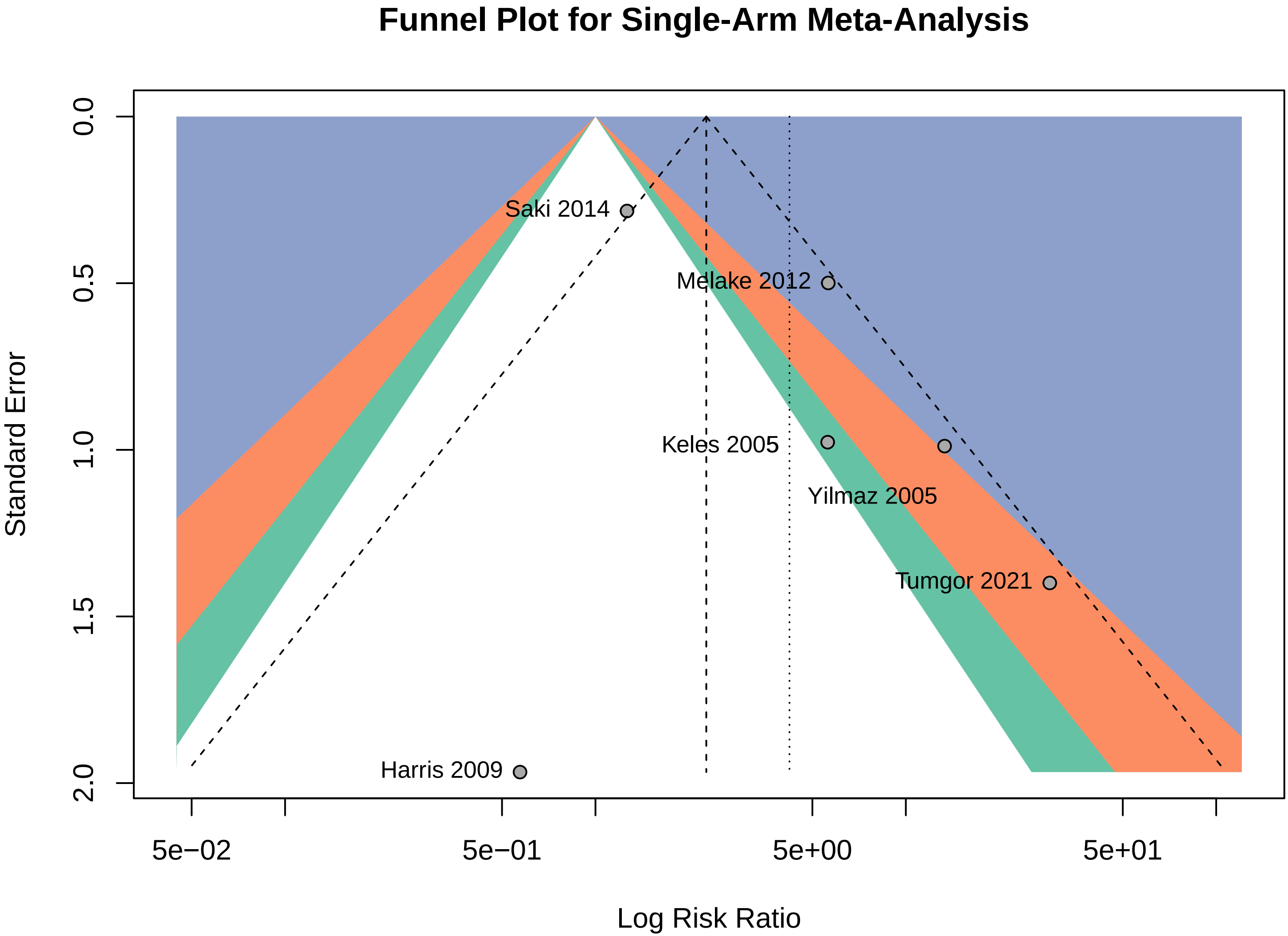
Funnel Plot for Single-Arm Meta-Analysis
Standard Error
0.0
0.5
1.0
1.5
2.0
Saki 2014
Melake 2012
Keles 2005
Yilmaz 2005
Tumgor 2021
Harris 2009
5e−02
5e−01
5e+00
5e+01
Log Risk Ratio

Funnel Plot _sensitivity

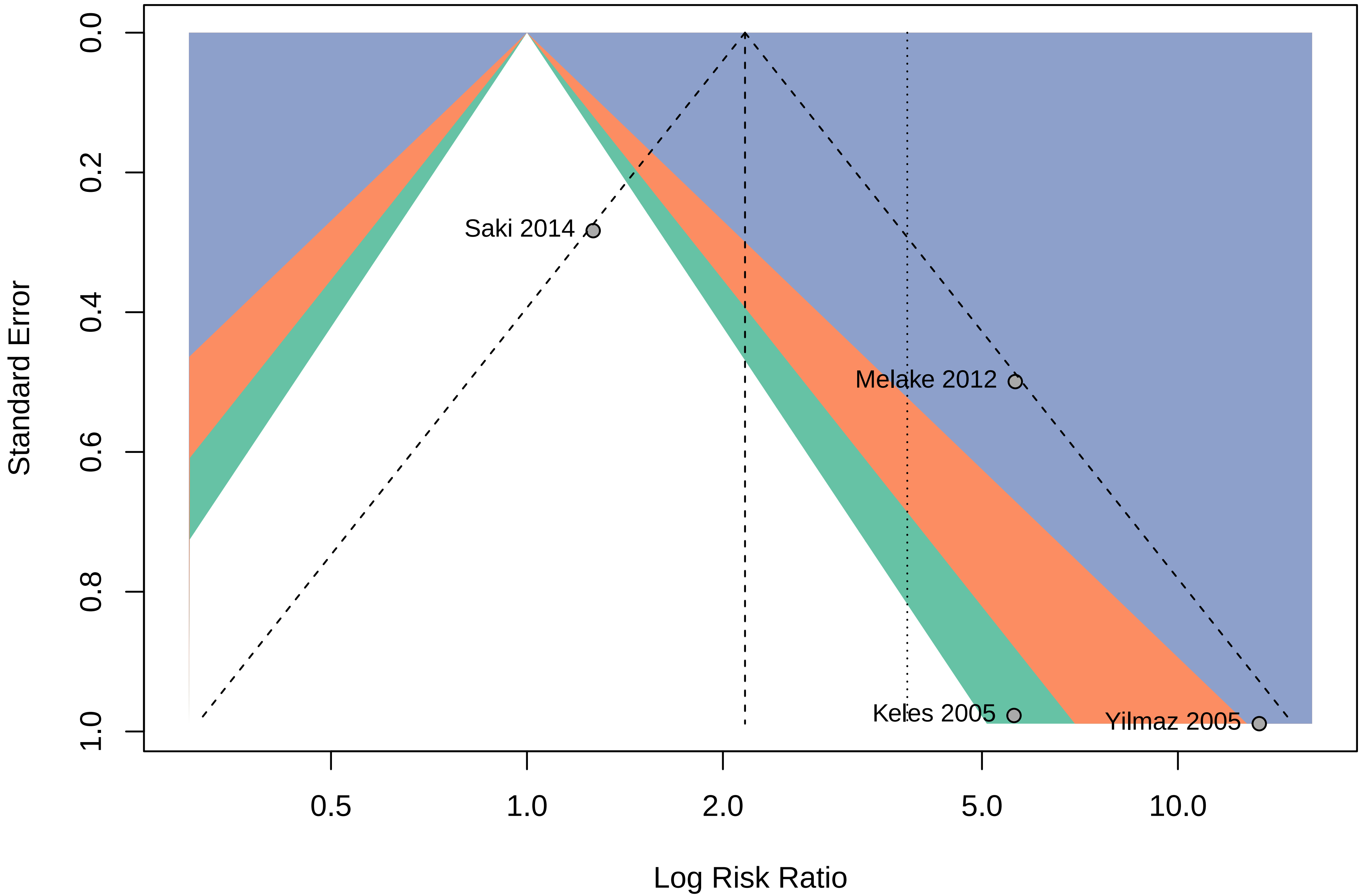

Sensitivity analysis of Egger ' s test for two-arm rate studies

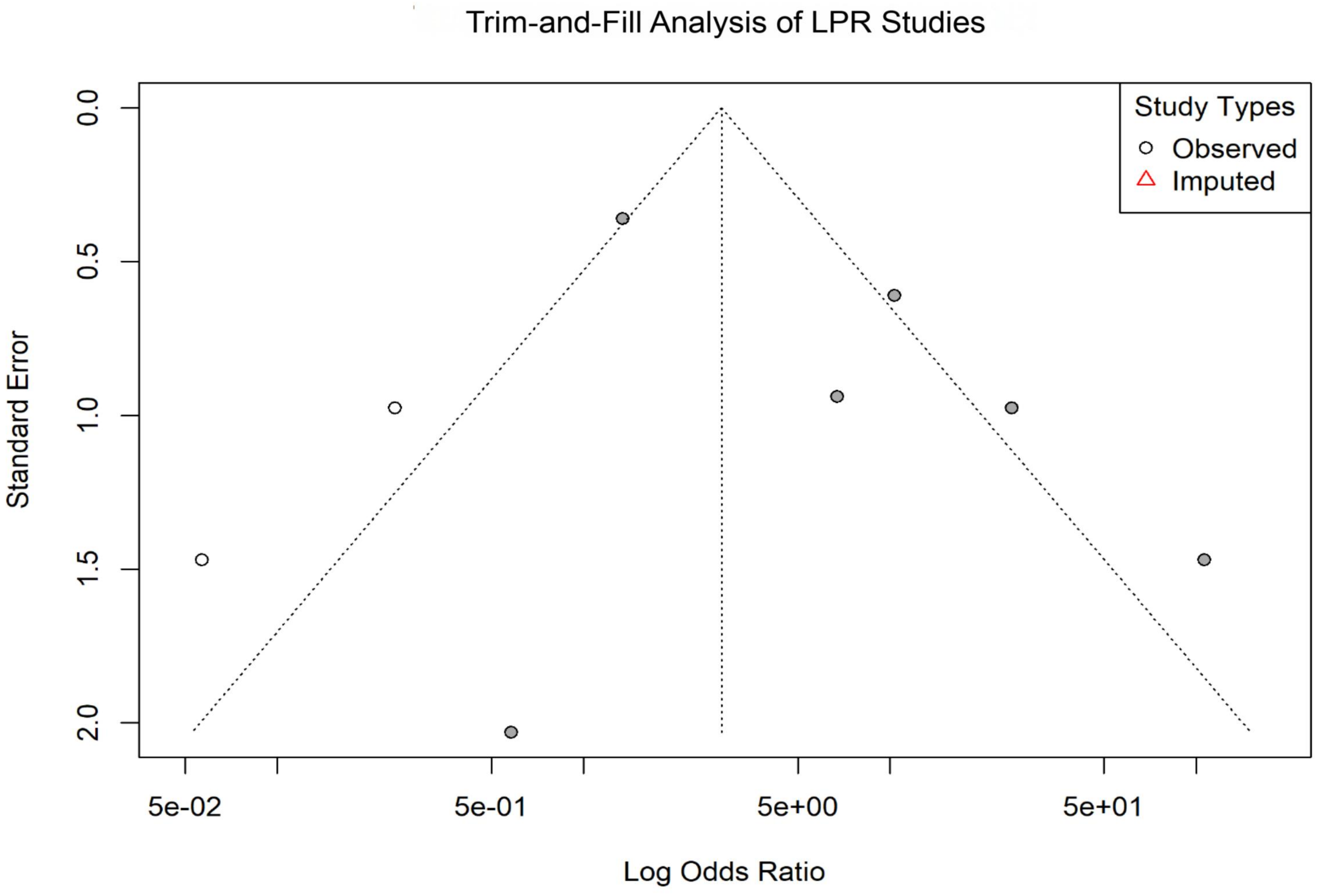
Trim-and-Fill Analysis of LPR Studies
Study Types
Observed
Imputed
Standard Error
0.0
0.5
1.0
1.5
2.0
5e-02
5e-01
5e+00
5e+01
Log Odds Ratio

Study	Experimental Events	Experimental Total	Control Events	Control Total	OR	95%−CI	Weight (common)	Weight (random)
Dearking 2012	638	8245	314	8245	2.12	[1.84; 2.43]	90.4%	36.7%
Shatz 2004	0	24	21	24	0.00	[0.00; 0.07]	6.6%	29.0%
Sagar 2020	2	49	10	49	0.17	[0.03; 0.80]	3.0%	34.3%
**Common effect model**		**8318**		**8318**	**1.92**	**[1.68; 2.20]**	**100.0%**	**.**
**Random effects model**					**0.14**	**[0.00; 374.81]**	**.**	**100.0%**

Heterogeneity: $I^2 = 92.7\%$, $\tau^2 = 8.9544$, $p < 0.0001$

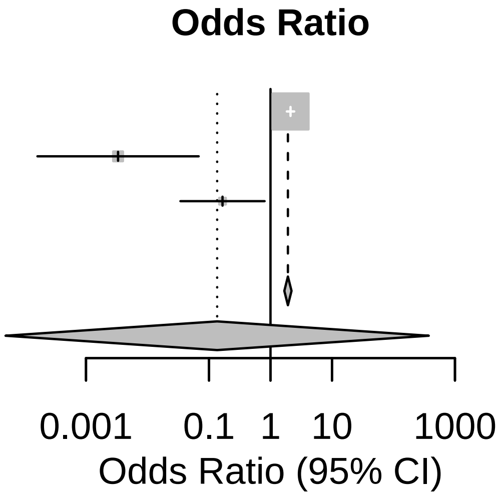

Meta-analysis of LPR improvement outcomes post-AT (Odds Ratio)

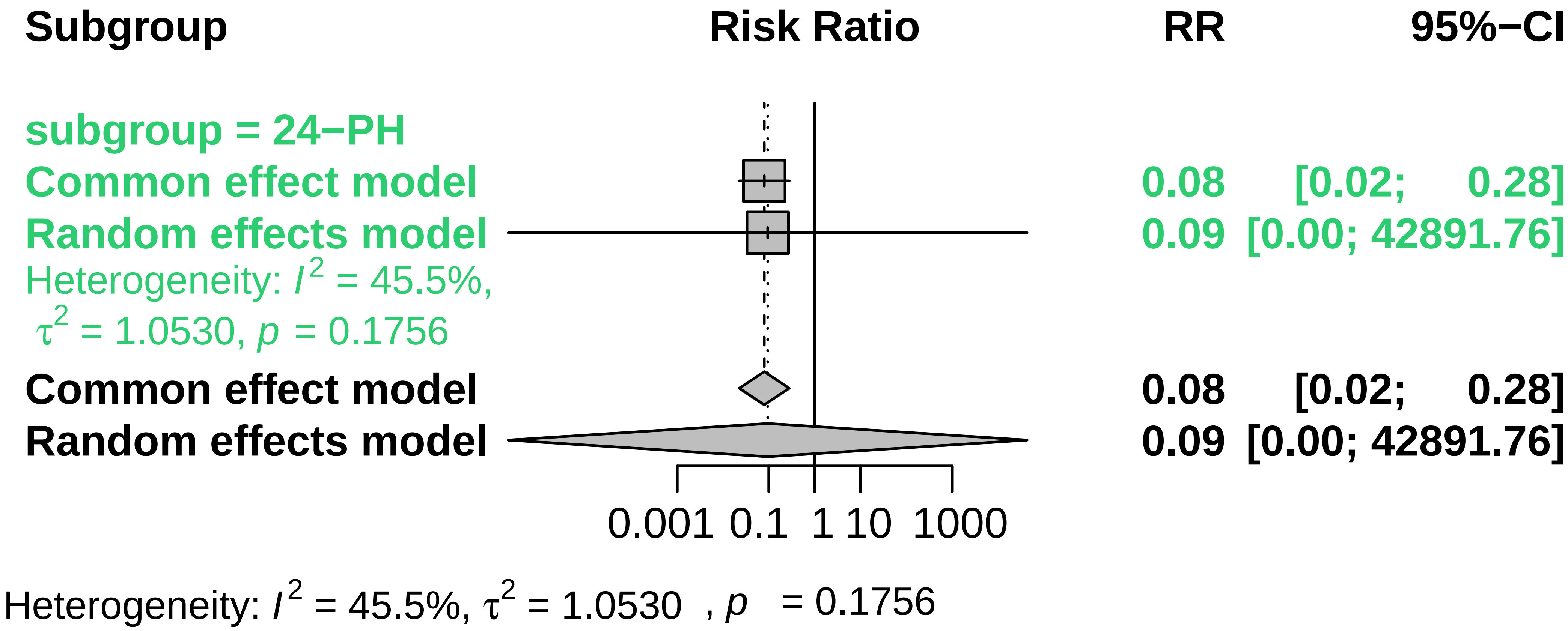

Subgroup meta-analysis of LPR improvement after AT (Risk Ratio)

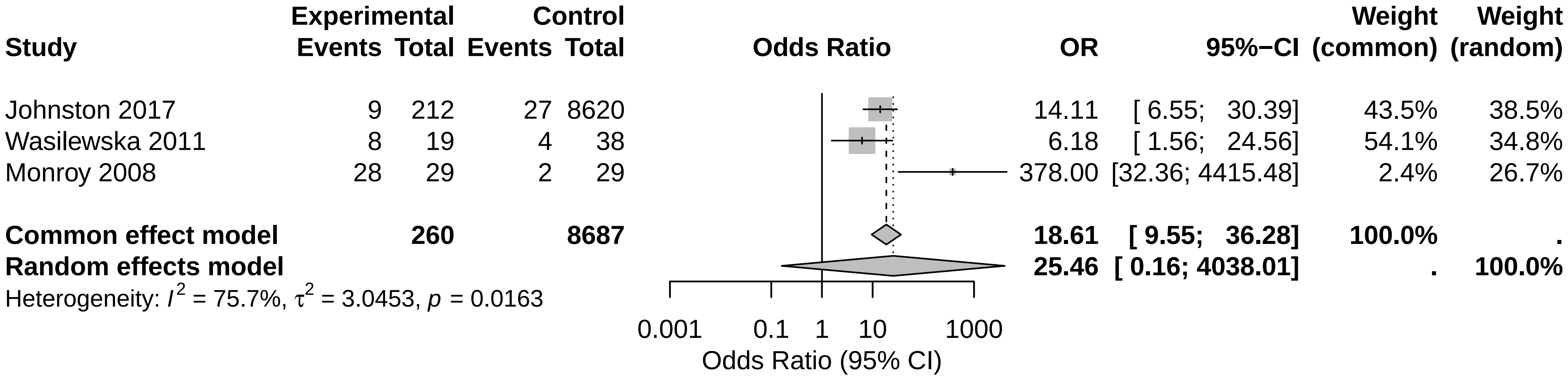

Meta-analysis of LPR incidence following revision AT surgery (Odds Ratio)

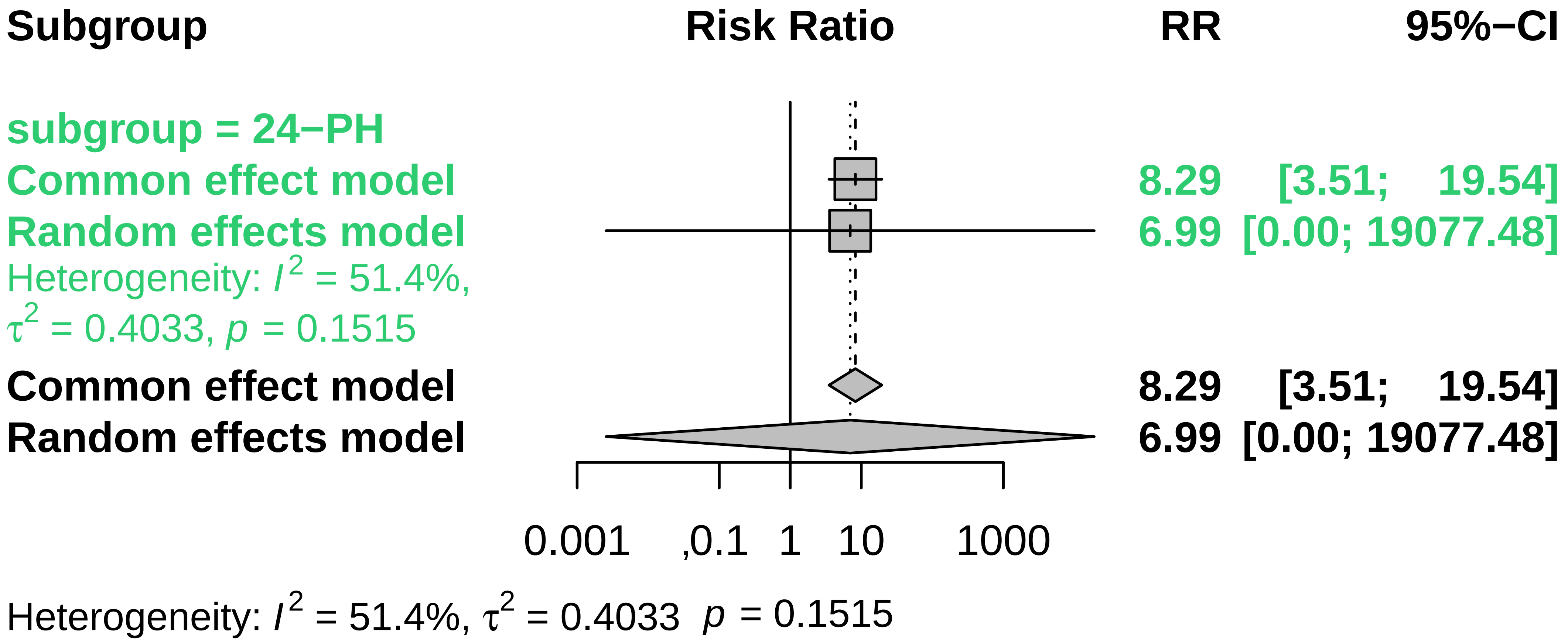

Stratified risk ratio analysis of recurrent LPR post-revision AT
